# Prevalence and Co-prevalence of Comorbidities among Patients with Type 2 Diabetes Mellitus in the MENA Region: A Systematic Review

**DOI:** 10.2174/1573399820666230731105704

**Published:** 2024-04-05

**Authors:** Samir Assaad Khalil, Sami Azar, Khadija Hafidh, George Ayad, Mohamed Safwat

**Affiliations:** 1 Department of Internal Medicine, Faculty of Medicine, Alexandria University, Alexandria, Egypt;; 2 Department of Internal Medicine, Faculty of Medicine, American University of Beirut, Beirut, Lebanon;; 3 Department of Diabetes and Endocrinology, Dubai Hospital, Dubai Health Authority, Dubai, United Arab Emirates;; 4 Regional Expert Input and Medical Education, EMEAC Region, MSD Egypt, Cairo, Egypt;; 5 Health Economics & Outcomes Research, CORE EEMEA, MSD United Arab Emirates, Dubai, United Arab Emirates

**Keywords:** Diabetes mellitus, type 2 diabetes, prevalence, comorbidities, complications, MENA region

## Abstract

**Aim:**

The management of type 2 diabetes mellitus is affected by the presence of comorbidities. This meta-analysis aimed to determine how likely it is for individuals with type 2 diabetes in the Middle East and North Africa (MENA) region to be living with additional chronic health conditions.

**Methods:**

We searched for studies published from January 2010 to December 2020 in the PubMed, Ovid MEDLINE®, Cochrane CENTRAL, Scopus, and Web of Science databases. Studies of adults with type 2 diabetes in the MENA region were included. We performed a random-effects meta-analysis of single proportions to calculate each comorbidity's overall prevalence/co-prevalence.

**Results:**

Statistically significant co-prevalence was detected at *p <* 0.01 for angina (pooled proportion: 0.24, 95% CI: 0.06, 0.49), cerebrovascular accident (pooled proportion: 0.16, 95% CI: 0.08, 0.26), coronary artery disease (pooled proportion: 0.25, 95% CI: 0.16, 0.35), coronary heart disease (pooled proportion: 0.05, 95% CI: 0.01, 0.12), peripheral vascular disease (pooled proportion: 0.19, 95% CI: 0.13, 0.26), hypertension (pooled proportion: 0.56, 95% CI: 0.43, 0.69), renal impairment (pooled proportion: 0.19, 95% CI: 0.10, 0.29), in addition to hyperlipidemia and overweight/obesity.

**Conclusion:**

There is evidence of co-prevalence of several comorbidities in patients with type 2 diabetes, highlighting the importance of enhancing communication among healthcare professionals to develop the optimal management plan for each patient.

## 1 INTRODUCTION

Diabetes is a serious global public health issue. According to some estimates, over one-third of the world's population is expected to be affected by this illness by the year 2050 [1]. In 2019, nearly 12.8% of the Middle East and North Africa (MENA) region's adult population had a diabetes diagnosis. The International Diabetes Federation (IDF) expects the prevalence rate in this region to rise to 15.7% by 2045, the second-highest increase among all IDF areas [2]. A recent systematic review and meta-analysis revealed a prevalence of type 2 diabetes mellitus (T2DM) of approximately 46 million individuals in the Middle East alone [3]. According to population-based studies, elderly T2DM patients commonly experience coexisting illnesses: most people with T2DM have at least one comorbidity, and some have multiple [4]. Ischemic heart disease, heart failure (HF), chronic kidney disease (CKD), chronic lung disease, hypertension, dyslipidemia, and obesity are the most common co-morbidities among these patients [5, 6]. Endocrinologists typically face challenges when treating T2DM patients with comorbidities as these conditions rarely occur in isolation and have the potential to interact [5].

As stated in a consensus report by the American Diabetes Association (ADA) and the European Association for the Study of Diabetes (EASD) on precision medicine in diabetes treatment options, choosing an anti-diabetic drug should be based on an individual's medical history and characteristics [7]. For example, metformin is unsuitable for patients with more advanced stages of renal insufficiency [4], and it is well-known that sulfonylureas increase the risk of hypoglycemia [8]. Dipeptidyl peptidase (DPP)-4 inhibitors and Glucagon-like Peptide (GLP)-1 receptor agonists have also been associated with an increased risk of pancreatitis [6]. Sodium–glucose co-transporter (SGLT_2_) inhibitors and GLP-1 receptor agonists are both reported to reduce cardiovascular disease (CVD) risk in patients with T2DM [9].

Several clinical investigations have indicated that patients with diverse comorbidities and etiologies respond differently to therapeutic strategies [7]. Key insights into the prevalence and nature of comorbidities in T2DM patients should help primary care providers better diagnose and treat individuals with multiple illnesses, allowing for more patient-centred risk assessments and tailoring of treatment. Comorbidity prevalence can assist policymakers in planning and structuring health services to meet the needs of an ever-growing population.

Thorough investigations into comorbidities in T2DM patients in the MENA region still need to be completed. This meta-analysis sought to discover how likely people with T2DM in the MENA are to have additional chronic conditions, such as hypertension, heart disease, stroke, and cancer. This is the first study to report on the prevalence and co-prevalence of such disorders in T2DM patients in this region; accordingly, our study can guide future research in this understudied area to prompt the optimization of T2DM care in the light of novel data on the burden and interplay of comorbidities.

## 2 METHODS

This study followed the Preferred Reporting Items for Systematic Reviews and Meta-Analyses (PRISMA) statement for the quality of reporting meta-analyses [10]. All steps were conducted in accordance with the Cochrane Handbook of Systematic Reviews and Meta-Analysis [11]. We used MeSH terms to search for studies published from January 2010 to December 2020 in the PubMed, Ovid MEDLINE®, EMBASE, Cochrane CENTRAL, Google Scholar, and Web of Science. The searches were repeated just before the final analyses, and further studies were retrieved for inclusion. The MeSH terms used are listed in the Supplementary Appendix.

The review included randomized and non-randomized clinical trials and observational studies (including epidemiological studies) that reported baseline characteristics with a minimum sample size of 50 patients with T2DM in the MENA region. Only English-language articles were included. The included studies reported the prevalence of at least one co-morbidity: Atherosclerotic cardiovascular disease (ASCVD), HF, CKD, hypertension, hyperlipidemia, or overweight/obesity.

The exclusion criteria were as follows: Studies before 2010, non-human or *in vitro* studies, abstract-only papers, studies with data not reliably extracted, duplicate or overlapping data, case reports, case series, and systematic reviews, studies that excluded patients with some of the co-morbidities of interest (ASCVD, HF, CKD, hypertension, hyperlipidemia, and overweight/obesity), or studies that reported type 1 or gestational diabetes.

The screening of retrieved search findings was performed by seven researchers, followed by conventional double screening by two independent reviewers.

### 2.1 Data Extraction

The extracted data included the article title, author(s), journal, publication date, country, setting, race, sample size, gender, mean age, glycemic control, diabetes treatment and duration, and co-morbidity prevalence.

### 2.2 Assessment of Risk of Bias

Three reviewers were involved in the quality assessment. They followed the PRISMA guidelines for study design, search protocol, screening, and reporting. The ROBINS-I tool and Newcastle-Ottawa Scale (NOS) checklists were used to value the selected RCTs, NRS, and observational studies concerning various aspects of the methodology and study process. Risk-of-bias plots were created through the Robvis online tool. A fourth reviewer resolved any discrepancies.

### 2.3 Statistical Analysis

A random effects meta-analysis of single proportions was carried out to calculate each co-morbidity's overall prevalence/co-prevalence. A generalized linear mixed model (GLMM) was also used for pooling. Specifically, a random intercept logistic regression model. The “Metaprop” function in the R software package “Meta” was used for the analysis.

Heterogeneity was assessed by the Chi-Square test (X^2^) and I-Square (I^2^) statistic was used for heterogeneity evaluation. Following the Cochrane Handbook for Systematic Reviews of Interventions 10, I^2^ was interpreted as follows: “0% to 40%: might not be important; 30% to 60%: May represent moderate heterogeneity; 50% to 90%: May represent substantial heterogeneity; 75% to 100%: Considerable heterogeneity. A meta-analysis was performed if the heterogenicity was> 75%.

## 3 RESULTS

A total of 202 studies were included in our review. Our search identified 15,001 studies, all retrieved from the specified databases (Supplementary Flow Chart 1); we removed duplicate records (n = 4,407) and screened the remaining records for ineligibility and/or inability to retrieve them. Upon assessment of the studies for the eligibility criteria, the reasons for exclusion were sample size less than 50 (n = 29), *in-vitro* studies (n = 7), case series (n = 4), and absence of comorbidities of interest (n = 3).

### 3.1 The Co-prevalence of CVD in Patients with T2DM

A forest plot was used to visualize the heterogeneity and pooled results. The outcome effect measure was expressed as a proportion. The diamond crosses the vertical line in the pooled estimate (random-effects model). It thus shows a statistically significant difference (*p <* 0.01) in the forest plot analysis of angina (pooled proportion: 0.24, 95% CI: 0.06, 0.49), cerebrovascular accident (pooled proportion: 0.16, 95% CI: 0.08, 0.26), coronary artery disease (CAD) (pooled proportion: 0.25, 95% CI: 0.16, 0.35), coronary heart disease (CHD) (pooled proportion: 0.05, 95% CI: 0.01, 0.12), heart disease (pooled proportion: 0.2, 95% CI: 0.14, 0.26), stroke (pooled proportion: 0.05, 95% CI: 0.04, 0.07), and peripheral vascular disease (PVD) (pooled proportion: 0.19, 95% CI: 0.13, 0.26). Further details are provided in Supplementary Materials Figs. (**S1-S7**).

However, there were no statistically significant differences in the cases of heart attack (pooled proportion: 0.04, 95% CI: 0.04, 0.05), at *p* =0.18), and myocardial ischemia (pooled proportion: 0.21, 95% CI: 0.2, 0.22, at *p* =0.48). Further details are provided in Supplementary Materials Figs. (**S8** and **S9**).

The statistical heterogeneity (I^2^) indicated the level of heterogeneity was 99% in the cases of angina, cerebrovascular accident, and coronary heart disease. The heterogeneity level was 100% in the case of coronary artery disease, 92% in heart disease, 96% in stroke, and 97% in peripheral vascular disease. As such, the studies were considered heterogeneously high. In contrast, the level of heterogeneity was 39% in the case of heart attack indicating low heterogeneity in the included studies. In the case of myocardial ischemia, the heterogeneity level was 0%, and as such the studies were not considered heterogeneous.

The funnel plot was used to assess the risk of publication bias in the included studies. High publication bias was shown as asymmetrical scatter in cases of cerebrovascular accident, CAD, stroke, and PVD. No risk of publication bias was observed in the case of CHD. However, the funnel plot are not useful when fewer than ten studies were included in an analysis. Further details are provided in Supplementary Materials Figs. (**S10**-**S18**).

### 3.2 The Co-prevalence of Hypertension in Patients with T2DM

According to the forest plot, the level of heterogeneity was 100%. As such the studies were considered heterogeneously high. In the pooled estimate (random-effects model), the diamond crossed the vertical line (pooled proportion: 0.56, 95% CI: 0.43, 0.69), thus showing a statistically significant difference at *p <* 0.01, as shown in Supplementary Materials Fig. (**S19**). The funnel plot showed a symmetrical scatter, indicating no risk of publication bias, as shown in Supplementary Materials Fig. (**S20**).

### 3.3 The Co-prevalence of Hyperlipidemia in Patients with T2DM

In the random effects model of hyperlipidemia, high cholesterol, high triglycerides, high LDL-C, and low HDL-C, the diamond crossed the vertical line indicating a statistically significant difference at *p <* 0.01 (pooled proportion: 0.6, 95% CI: 0.51, 0.68, pooled proportion: 0.37, 95% CI: 0.33, 0.42, pooled proportion: 0.43, 95% CI: 0.38, 0.48, pooled proportion: 0.41, 95% CI: 0.33, 0.49 and pooled proportion: 0.5, 95% CI: 0.42, 0.57, respectively). The level of heterogeneity was 99% in the cases of hyperlipidemia, high cholesterol, high LDL-C, and low HDL-C and 98% in the case of high triglycerides indicating high heterogeneity in the included studies. Further details are provided in Supplementary Materials Figs. (**S21-S25**).

The funnel plot showed a high risk of publication bias in the cases of high cholesterol, high triglycerides, high LDL-C, and low HDL-C. However, it showed no risk of bias in the case of hyperlipidemia. Further details in Supplementary Materials Figs. (**S26-S30**).

### 3.4 The Co-prevalence of Renal Dysfunction in Patients with T2DM

The forest plot of end-stage renal disease (ESRD)and renal impairment indicated that the level of heterogeneity for both was 98%. As such, the studies were considered heterogeneously high. The random effects model showed statistically significant differences at *p <* 0.01 (pooled proportion: 0.05, 95% CI: 0.01, 0.12, pooled proportion: 0.19, 95% CI: 0.10, 0.29, respectively). Further details are provided in Supplementary Materials Figs. (**S31** and **S32**).

The funnel plot was not valuable for analysis of the risk of bias for ESRD or renal impairment. Further details are provided in Supplementary Materials Figs. (**S33** and **S34**).

### 3.5 The Co-prevalence of Overweight/ Abdominal Central Obesity in Patients with T2DM

The forest plot of overweight and obesity indicated that the level of heterogeneity was 98% and 99%, respectively. Accordingly, the studies were considered heterogeneously high. The random-effects model showed statistically significant differences at *p <* 0.01 (pooled proportion: 0.36, 95% CI: 0.39, 0.42, pooled proportion: 0.7, 95% CI: 0.63, 0.77, respectively). Further details are provided in Supplementary Materials Figs. (**S35** and **S36**).

The funnel plot was not useful for the analyzing of the risk of bias for central abdominal obesity but showed a high risk of publication bias in the case of overweight. Further details are provided in Supplementary Materials Figs. (**S37** and **S38**).

### 3.6 The Prevalence of Glycemic Control in Patients with T2DM

The forest plot of HbA1c and macro-albumin indicated that the level of heterogeneity was 100% for both. Accordingly, the studies were considered heterogeneously high. The random-effects model showed statistically significant differences at *p <* 0.01 (pooled proportion: 0.4, 95% CI: 0.27, 0.53, pooled proportion: 0.24, 95% CI: 0.03, 0.55, respectively). Further details are provided in Supplementary Materials Figs. (**S39** and **S40**).

The funnel plot was not useful for analysis of the risk of bias for macro-albumin but showed no risk of publication bias in the case of HbA1c. Further details are provided in Supplementary Materials Figs. (**S41** and **S42**).

## 4 DISCUSSION

Most current disease management guidelines are designed to address individual health conditions, so several co-morbidities pose challenges for physicians when deciding the patient’s management plan and the choice of antidiabetic drugs while balancing the risks and benefits of polypharmacy [12]. For patients, a diagnosis of several co-morbidities results in increased medication use, billing burden, costs, and the risk of depression alongside a decline in treatment adherence and quality of life [4, 13, 14].

The goal of this study was to determine how common it is for people with type 2 diabetes to be living with other chronic conditions, such as heart disease or stroke. T2DM represents a growing burden in the MENA region, accounting for 4.3% of all Disability Adjusted Life-Years (DALYs) in 2015, up from 2.5% in 2000 [15]. In 2010, there were 285 million T2DM patients; by 2040, the number of patients is expected to reach 642 million [16].

Many of these co-morbidities often have a similar or interacting pathophysiological mechanism, a causal factor in the significant co-prevalence. In the published literature, the rate of co-prevalence varied among the studies, which may be attributed to differences in population characteristics, case definition, study design, and settings.

Our findings revealed a statistically significant co-prevalence of several co-morbidities among T2DM patients in the MENA region, including angina, cerebrovascular accident, CAD, CHD, heart disease, peripheral vascular disease, hypertension, hyperlipidemia, abnormal lipid profile, ESRD, renal impairment, overweight, central abdominal obesity, stroke, poor glycemic control presenting as HbA1c abnormal levels, and macro albumin abnormal levels. However, no statistically significant co-prevalence was found in the case of a heart attack. Higher prevalence proportions were associated with central abdominal obesity, overweight, hypertension, and abnormal lipid profile, where obesity co-prevalence with T2DM was estimated to be as high as 70% and co-prevalence of high cholesterol levels to be 60%.

In a previous retrospective study by Iglay *et al*., the most common co-morbidities in T2DM patients were hypertension (82.1%), overweight/obesity (78.2%), hyperlipidemia (77.2%), chronic kidney disease (24.1%), and CVD (21.6%), which is in line with our findings [5]. Hypertension and metabolic syndrome were the most commonly detected co-morbidities in another study by Hermans *et al*., which also reported that most T2DM patients had at least four co-morbid conditions [17].

In our study, CAD, heart disease, and angina had the greatest co-prevalence among the cardiovascular comorbidities, with an overall prevalence of 25%, 20% and 24%, respectively, while the least observed cardiovascular co-morbidities were stroke and CHD, each with an overall prevalence of 5%. Similar results were described in a major systematic review and meta-analysis in 2018, where 32.2% of T2DM patients had CVD, of which CAD was the most common form (21.2%). Fewer cases of stroke were recorded than any other injury. According to that study, T2DM patients died from CVD at a rate of roughly 50.3% [18].

Similarly, the poor glycemic control we observed was also detected in a large observational analysis of 66,088 T2DM patients in developing countries. Despite recent breakthroughs in T2DM therapies, many patients cannot adhere to treatment plans because they need to receive adequate organized instruction or sufficient face-to-face contact from healthcare experts [19].

The observed prevalence of renal co-morbidities has also been reported extensively in the literature concerning diabetic nephropathy (DN), a common complication of T2DM and considered an independent risk factor for developing ESRD and CVD in T2DM. Moreover, T2DM patients with high blood pressure were found to have a higher risk of developing diabetic nephropathy in a prior study [20]. Studies also have shown that T2DM patients had a higher-than-average prevalence of obesity and an aberrant lipid profile, which are known to increase the risk of CVD [18, 21, 22]. These findings demonstrate the intricate interplay of several co-morbidities in diabetic patients, necessitating treatment strategies that are appropriate and comprehensive.

Another challenge is the need for appropriate communication and coordination between different healthcare providers responsible for the management of co-existing conditions [23]. This is essential to facilitate utilizing the existing evidence in the literature and cross-referencing with the existing guidelines in order to develop new guidance on managing co-morbidities that balances the risk and benefits while considering patients’ compliance [24, 25].

The main strength of our study is that this meta-analysis is considered the first to investigate the co-prevalence of co-morbidities with T2DM in the MENA region, and it could help direct future guidance and decision-making for T2DM patients with multiple health conditions.

The limitations of our research include the increased heterogeneity among most of the included studies and the increased publication bias. Our analysis only included data from adult T2DM patients; thus, the conclusions cannot be applied to children, adolescents, or those with secondary diabetes. Furthermore, interaction effects were not considered, and subanalyses of different comorbidities were not performed. This is primarily because data on co-prevalence available in the existing literature is still being determined. In addition, there needs to be more research methodology and reporting methods in studies from the MENA region. Adopting more institutionalized and rigorous protocols will allow researchers to utilize synthesized evidence and generate informed practice guidelines and policies.

## CONCLUSION

The most common condition in patients with T2D included were central obesity (70%), hyperlipidemia (60%), hypertension (56%), low HDL-C (50%), high triglycerides (43%), and high LDL-C (41%). The least common conditions were coronary heart disease, stroke and ESRD (5% each) and heart attack (4%). The analysis also revealed that 40% of subjects had adequate glycemic control.

In summary, there is evidence of the co-prevalence of T2DM with angina, cerebrovascular accident, CAD, CHD, heart disease, PVD, hypertension, hyperlipidemia, abnormal lipid profile, ESRD, renal impairment, overweight, central abdominal obesity, stroke, abnormal HbA1c levels, and abnormal macro albumin levels in the MENA region. These findings should guide physicians during clinical decision-making, where additional care should be directed towards managing present co-morbidities and preventing their progression. Our research also calls for enhancing communication between healthcare professionals to reach the optimal management plan for T2DM patients. Additionally, this research should act as a gateway for further specialized research in this area, where structured data is required to guide the development of more effective therapeutic strategies.

## Data Availability

The data and supportive information are available within the article.

## LIST OF ABBREVIATIONS

T2DM: Type 2 Diabetes Mellitus
DN: Diabetic Nephropathy
HF: Heart Failure
CKD: Chronic Kidney Disease
IDF: International Diabetes Federation
DPP: Dipeptidyl Peptidase
GLP: Glucagon Like Peptide
CVD: Cardiovascular Disease

## SUPPLEMENTARY MATERIALS

Flow chart 1: Identification of studies *via* databases and registers Figure 1: Forest plot for Angina Figure 2: Forest plot for cerebrovascular accident Figure 3: Forest plot for coronary artery disease Figure 4: Forest plot for coronary heart disease Figure 5: Forest plot for heart disease Figure 6: Forest plot for stroke Figure 7: Forest plot for peripheral vascular disease Figure 8: Forest plot for heart attack Figure 9: Forest plot for myocardial ischemia Figure 10: Funnel plot for cerebrovascular accident Figure 11: Funnel plot for coronary artery disease Figure 12: Funnel plot for stroke Figure 13: Funnel plot for peripheral vascular disease Figure 14: Funnel plot for coronary heart disease Figure 15: Funnel plot for angina Figure 16: Funnel plot for heart attack Figure 17: Funnel plot for heart disease Figure 18: Funnel plot for myocardial ischemia Figure 19: Forest plot for high blood pressure Figure 20: Funnel plot for high blood pressure Figure 21: Forest plot for hyperlipidemia Figure 22: Forest plot for high cholesterol Figure 23: Forest plot for high LDL Figure 24: Forest plot for low LDL Figure 25: Forest plot for high triglycerides Figure 26: Funnel plot for high cholesterol Figure 27: Funnel plot for high triglycerides Figure 28: Funnel plot for high LDL Figure 29: Funnel plot for low LDL Figure 30: Funnel plot for hyperlipidemia Figure 31: Forest plot for ESRD Figure 32: Forest plot for renal impairment Figure 33: Funnel plot for ESRD Figure 34: Funnel plot for renal impairment Figure 35: Forest plot for overweight Figure 36: Forest plot for central abdominal obesity Figure 37: Funnel plot for central abdominal obesity Figure 38: Funnel plot for overweight Figure 39: Forest plot for HbA1c Figure 40: Forest plot for macro albumin Figure 41: Funnel plot for HbA1c Figure 42: Funnel plot for macro albumin Supplementary material is available on the publishers Web site along with the published article.
